# Nanoscale reference and test materials for the validation of characterization methods for engineered nanomaterials — current state, limitations, and needs

**DOI:** 10.1007/s00216-024-05719-6

**Published:** 2025-01-04

**Authors:** S.-L. Abram, I. Tavernaro, L. J. Johnston, S. Zou, U. Resch-Genger

**Affiliations:** 1 Division Biophotonics, Federal Institute for Materials Research and Testing (BAM), Richard-Willstaetter-Str. 11, 12489 Berlin, Germany; 2 Metrology Research Centre, National Research Council Canada (NRC), 100 Sussex Drive, Ottawa, ON K1A 0R6 Canada

**Keywords:** Engineered nanomaterials, Nanoscale reference materials, Interlaboratory comparisons, Traceability, Standardization, Regulations

## Abstract

The rational design of engineered nanomaterials (NMs) with improved functionality and their increasing industrial application requires reliable, validated, and ultimately standardized characterization methods for their application-relevant, physicochemical key properties such as size, size distribution, shape, or surface chemistry. This calls for nanoscale (certified) reference materials (CRMs; RMs) and well-characterized reference test materials (RTMs) termed also quality control (QC) samples, assessed, e.g., in interlaboratory comparisons, for the validation and standardization of commonly used characterization methods. Thereby, increasing concerns regarding potential risks of NMs are also addressed and the road for safe and sustainable-by-design concepts for the development of new functional NMs and their use as nanomedicines is paved. With this respect, we will provide an overview of relevant international standardization and regulatory activities, definitions, and recommendations on characterization methods and review currently available organic or inorganic nanoscale CRMs, RMs, and RTMs, including their characterization or certification. In addition, we will highlight typical applications to streamline the regulatory approval process and improve manufacturability including the special challenges imposed by the colloidal nature and sometimes limited stability of NMs. Subsequently, we will critically assess the limitations of currently available nanoscale RMs and RTMs and address the gaps to be filled in the future such as the availability of NMs that come with reference data on properties other than commonly addressed particle size, such as surface chemistry or particle number concentration, or more closely resemble commercially available formulations or address application-relevant matrices.

**Graphical Abstract:**

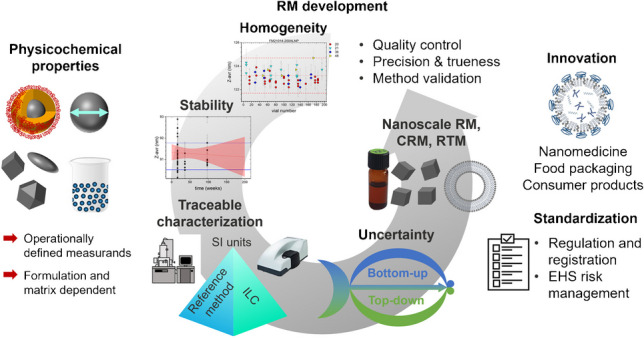

## Introduction

**Fig. 1:**
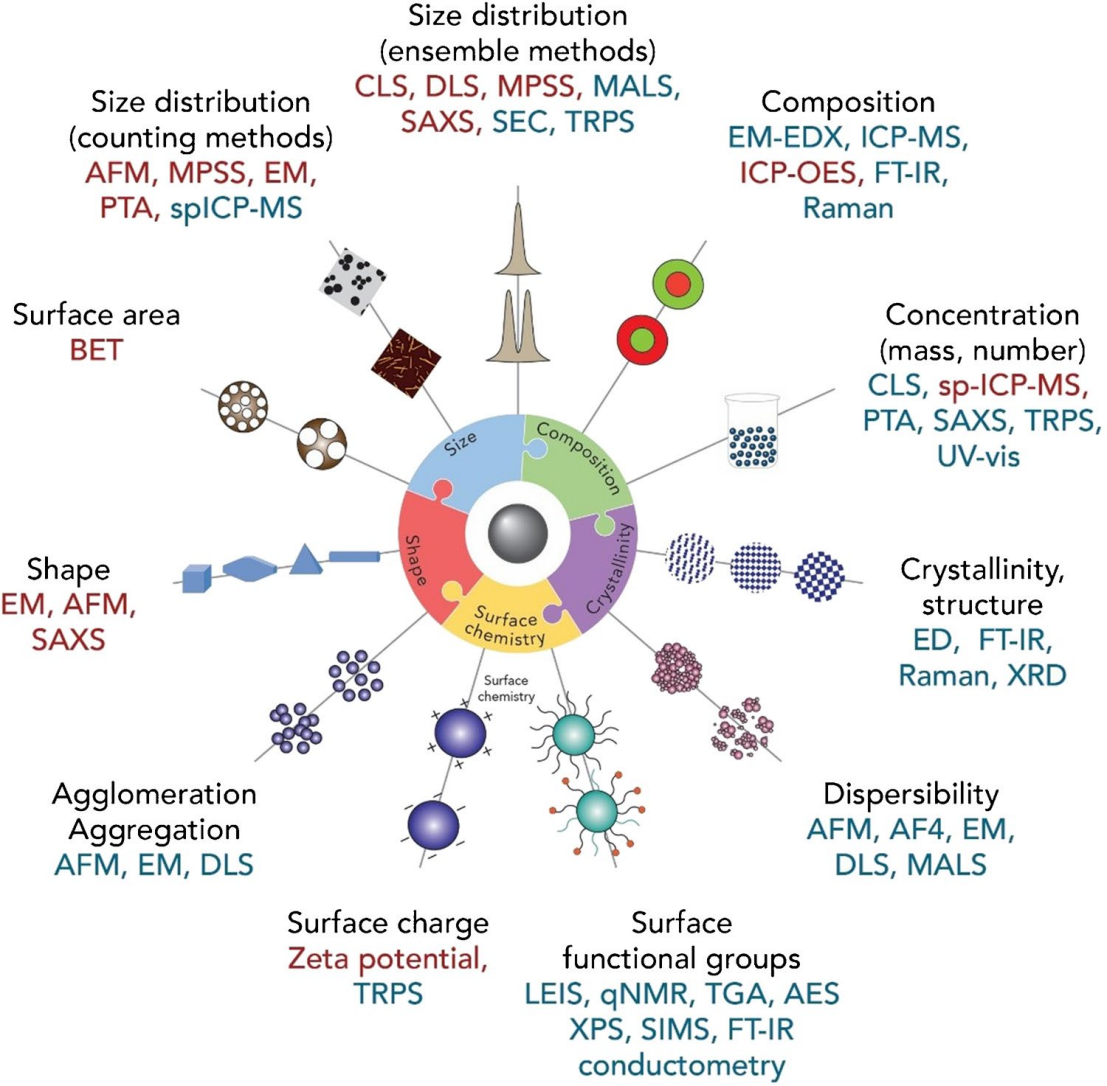
Properties required for characterization of nanomaterials and commonly used methods applicable to a range of samples are presented. Methods that have been used to certify CRMs are in red and other methods in blue. Many of the methods can be classified as either beam techniques (e.g., EM, XPS, SAXS, FT-IR, Raman) or methods that require samples digestion (ICP-MS, ICP-OES). This figure was adapted from reference [3]

Engineered nanomaterials (NMs) are currently widely used in different application areas, ranging from consumer products over medical diagnostics, drug delivery, sensing, catalysis, energy storage and conversion to opto-electronics, and information storage [1, 2]. The broad diversity of application-relevant inorganic, organic, and hybrid NMs imposes considerable challenges on their characterization compared to most molecular compounds, where the determination of the chemical structure, composition, and concentration is commonly sufficient and analytical methods targeting these features even in complex matrices are frequently at hand. Properties that determine NM functionality include particle size and shape, size and shape distributions, surface charge and chemistry, composition (especially for core–shell materials), crystal phase, dispersibility, agglomeration/aggregation state, and particle number concentration (Fig. 1), although typically a subset of these features will be required for specific materials and applications [3–6]. These features, which can be also affected by the NM matrix, control the interaction of NMs with their surroundings and their stability as well as their potential risk to human health and the environment [7]. Depending on the desired NM end use, also optical, electronic, mechanical, magnetic, or catalytic properties have to be assessed [4]. The main parameters (composition, size distribution, shape, surface charge and chemistry, specific surface area) required for identifying a nano-object in the context of risk assessment have been summarized in an ISO technical report [8]. For medical applications or NM safety assessment, additionally, features such as endotoxin content, loading of active ingredients, potential for inflammation and immunosuppression [9, 10], or exposure, fate, transformation, and accumulation [11] are important. To ensure proper NM functionality and quality assurance and control during production, an adequate and reliable characterization of all these properties is essential. This facilitates the batch-to-batch comparison of NMs from one or several suppliers and ensures meaningful risk assessment data guaranteeing the safe use of engineered NMs throughout their life cycle. However, at present, many studies do not provide such an adequate NM characterization. This frequently prevents general conclusions across multiple studies and reduces the value of many toxicity studies [12], thereby hampering safe and sustainable-by-design concepts (SSbD).

These stringent requirements on NM characterization call for the development of well-characterized (certified) reference materials (CRMs; RMs) and representative test materials (RTMs) for establishing typical measurement techniques and verifying results (Fig. 1) in different laboratories and to support method standardization. Such nanoscale RMs, providing benchmark values, allow users to test and validate instrument performance and measurement protocols and to assess laboratory competence, thereby providing the backbone for comparable and reliable measurements in nanotechnology that facilitate the application of NMs, especially in strongly regulated areas such as medical diagnostics and therapy [9, 13, 14]. RMs can also strongly support and speed up the development of the next generation of more sustainable and eco-friendly NMs utilizing SSbD concepts [15].

In this review, we will first provide an overview of international standardization and regulatory activities including recommendations on NM characterization measurements, different types of nanoscale RMs and RTMs, their development and certification, and recent interlaboratory comparisons (ILCs) supporting the validation and standardization of the relevant characterization methods. In this context, definitions of NMs that vary between different countries will be summarized and complicate regulatory approval for NMs, the metrological hierarchy of RMs, and the underlying requirements on CRMs, RMs, and RTMs will be addressed to lay the foundation for judging the current gaps in the availability of RMs and RTMs. Finally, future needs will be critically emphasized considering measurement challenges imposed by the need to characterize application-relevant NMs in a broad variety of complex matrices in different fields of application such as nanomedicines, consumer products, and food.

## Standardization in nanotechnology — organizations and scope

Several international organizations develop standards for NM characterization based on a consensus approach involving manufacturer, consumer, and regulator stakeholders. These include the International Organization for Standardization (ISO), the International Electrotechnical Commission (IEC), ASTM International, the European Committee for Standardization (CEN), and the International Pharmaceutical Regulators Program (IPRP). Also, many national organizations develop standards or adopt international ones for local use. The timeline for developing a standard protocol for a characterization method with a clear and often narrow scope is typically on the order of 2–4 years. The protocol may be supported by validation data from an international ILC involving qualified labs. The fragmentation of efforts and the different approaches of the various organizations unavoidably lead to some overlap and duplication. National metrology institutes (NMIs) frequently participate in these standardization efforts. NMI competence in selected areas is maintained through participation in so-called key comparisons organized by the International Bureau of Weights and Measures (BIPM) coordinating NMI activities; these NMI comparisons ensure competence in specific measurement methods, which lays the basis for the production of RMs and for the development of standards and guidelines. Especially relevant for the nanotechnology area is a new task group on particle metrology within the BIPM Consultative Committee on Amount of Substance (CCQM), which will identify activities to be examined over the next 10 years, including so-called pilot studies, key comparisons, and cooperative research projects.

Other relevant organizations include the International Union of Pure and Applied Chemistry (IUPAC) and the Organization for Economic Cooperation and Development (OECD). IUPAC contributes to the standardization of chemical nomenclature and produces recommendations for terminology and reliable measurement methods. IUPAC facilitates research activities by supplying technical reports, books, and databases, with several recent examples covering analytical methods for characterizing NMs as produced and in complex media as well as for nanosafety [3, 7, 16, 17]. OECD develops test guidelines and guidance documents that address issues related to nanosafety, thereby targeting the need for standardized methods to characterize NMs and understand their effect on environmental species and human health. The OECD mutual acceptance of data policy ensures that data generated by one member country shall be accepted in other member countries. Also, several international large multi-center collaborative projects are running that involve partners from NMIs, academia, and industry that address NM metrology. Examples include the recently started European Partnership on Metrology (EMP) projects *Metrology for Innovative Therapeutics* (MetrINo) and *Standardised Measurements of Surface Functionalities on Nanoparticles* (SMURFnano). To broaden project impact, these projects often involve non-EU partners either directly or via the Scientific Advisory Board. Another example is the US Nanotechnology Characterization Laboratory (NCL) which aims to accelerate the progress of nanomedicine by providing preclinical characterization and safety testing of nanoparticles. It is a collaborative effort between the National Cancer Institute, the US Food and Drug Administration (FDA), and the National Institute of Standards and Technology (NIST).

## NM definitions and regulatory approval of NMs

The regulatory approval for NMs is currently complicated by nano definitions, which vary considerably depending on the organization and whether the definition is meant for general technical or regulatory use, or safety testing before the approval of NMs or nano-enabled products in, e.g., consumer products or nanomedicines. Most international organizations including ISO, IEC, and OECD define NMs as materials with at least one dimension on the nanoscale, typically from 1 to 100 nm; some also refer to the presence of novel properties not observed for bulk materials [18]. These general definitions are, however, not suitable for regulatory purposes, which often have specific requirements for the safety testing of NMs [19]. It is therefore important to specify (i) which types of NMs are covered, e.g., engineered, manufactured, or incidental NMs^1^ (ii) the acceptable method(s) for evaluating NM size; and (iii) how to deal with agglomerates or aggregates, i.e., whether they are ignored or counted as single particles or whether individual particles within the agglomerate are counted. In addition, a threshold based on mass, number, or volume for the amount of material that must be within the nanoscale range must be provided. Standard methods are still lacking to allow measurements of the thresholds specified in some definitions.

Currently, the approaches used by various countries and jurisdictions considerably vary as expected since the regulatory needs will depend on the regulator and application area. Some countries adopt the general ISO definition, and some have specific regulatory definitions that identify additional factors, but have different types of thresholds, and typically fail to specify methods to use for particle counting and dealing with agglomerates and aggregates. For example, the revised EU definition has a 50% by number threshold, whereas in the USA, a mass-based threshold is used [19]. Hence, a material can be classified as nano in some jurisdictions but not in others, creating a difficult situation for global companies that aim to sell NMs or nano-enabled products to a wide range of markets. A useful overview of the current regulatory definitions for NMs in different jurisdictions can be found in a recent summary in *Nature Nanotechnology* [19]. This article also proposes a naming convention for NMs that includes the name of the material, its origin, how particles are counted, and the threshold for nano classification. Related papers indicate how the current EU definition can be implemented [20, 21].

## Nanoscale CRMs, RMs, and RTMs — definitions, quality criteria, and traceability

The validation and standardization of NM characterization methods can be achieved by cross method comparisons, ILCs, or the usage of NMs with reliably known physicochemical properties. For the latter, simplest approach, CRMs and RMs play a crucial role by serving as benchmarks that ensure the accuracy and comparability of measurements and tests and help laboratories by providing known precision and bias values. Thereby, laboratories can verify the reliability of their in-house methods, which is essential for regulatory approval and industry acceptance [13, 22]. The concept of RTMs (sometimes referred to as quality control (QC) samples) was initially developed for toxicity testing, where extensive homogeneity testing is either ethically not acceptable, not feasible, or partially unnecessary. RTMs can also be used for the measurement of physicochemical properties and can serve as benchmarks if they have been, e.g., characterized in an ILC focussed on such measurements which require homogeneity studies and at least short-term stability studies and sometimes long-term stability monitoring. If the homogeneity and stability of these RTMs were determined for one property, the materials may then be considered stable and homogeneous for other parameters and therefore fit-for-purpose as, for example, those used in the OECD testing program.

CRMs, RMs, and especially RTMs should ideally be provided with a scope for their use and an in-depth description of the measurement method(s) utilized for their characterization, including instrumentation, instrument calibration, and uncertainties of the provided properties as well as storage conditions, shelf life, and a standard operation procedure (SOP) for use [23].

### Definitions, metrological hierarchy, and quality criteria

The gold standard for RMs is a CRM. A CRM is a material characterized by a metrologically valid procedure for one or more specified properties, accompanied by a certificate that provides the value of the specified property, its associated uncertainty, and a statement of metrological traceability [24]. Metrological traceability is defined as a property of a measuring result whereby the result can be related to a reference through a documented unbroken chain of calibrations, each contributing to the measurement uncertainty [25]. Typically, traceability to SI units (Système international d’unités = International System of Units) is established during the certification procedure, commonly via the required calibration of the instruments used for determination of the certified property. The reference can be a definition of a measurement unit through its practical realization or a measurement procedure including the measurement unit or a measurement standard. Although most RM producers use the term CRM, the NIST uses the term standard reference material (SRM, a registered trademark) for a material that meets the ISO CRM requirements and ERM is similarly used by EC-JRC. An RM is defined as a material that is sufficiently homogeneous and stable with respect to one or more specified properties and has been established to be fit for its intended scope in a measurement process [24]. Properties can be quantitative or qualitative and the uses may include instrument calibration, assessment of a measurement procedure, assignment of property values, and quality control. Note that a single RM cannot be used for both calibration and validation of results in the same measurement procedure. A measurement report is typically provided for an RM, but neither an uncertainty estimate nor traceability is required. Given the diversity of methods and materials and the time and effort spent on generating CRMs, there is considerable room and need for RMs and RTMs that fulfill the requirements for amount of material, batch homogeneity, and stability but do not have traceable property values. Frequently, CRMs and RMs, which are currently developed almost exclusively by NMIs or the European Commission Joint Research Centre (EC-JRC), come with additional, so-called informative or informal data on other properties obtained with a lower level of validation but useful for the end user. Both CRMs and RMs are available for purchase from the organization producing the materials and are in some cases also available from companies licensed to sell the materials. Since there are currently a limited number of nanoscale CRMs and RMs available, some studies have not followed the requirements outlined above for the use of such materials. An example is provided by an Au NP RM released by NIST, which did not have certified property values, but, in the absence of suitable CRMs, was used for calibration and method validation studies [26].

In addition, a variety of RTMs have been developed in large collaborative projects or ILCs. RTMs typically do not come with an uncertainty budget of the respective property or properties addressed in the ILC and are provided with less documentation than CRMs and RMs. However, for some RTMs, detailed characterization reports are available. For example, the EC-JRC RTMs [27] consist of an initial set of NMs from industrial sources, which were selected for use in a large OECD project on Safety Testing of Representative Manufactured NMs, which assessed the suitability of existing OECD methods for physicochemical characterization and toxicological testing [28]. The homogeneity and stability of these materials were determined for one property, and the materials were then considered stable and homogeneous with respect to other parameters and therefore fit-for-purpose for the OECD testing program. Studies using these RTMs have resulted in the revision of some OECD test guidelines and ongoing projects tackle the development of others [29]. The EC-JRC repository currently includes over 30 NMs, most of which are still available upon request and some of which have detailed characterization reports. It should be noted that although these materials have been very useful for toxicological tests which typically have a significant variability, they are not suitable for the measurement or validation of physicochemical properties (contrary to RTMs derived from ILCs focusing on physicochemical measurements as mentioned in a previous section). Other large collaborative programs and consortia have developed test materials for their internal use, which are, however, often not available to other laboratories and have had variable levels of homogeneity and stability testing. However, in some cases, they may provide useful QC samples for validation of consistent instrument performance.

Table 1 provides an overview of the nanoscale CRMs and RMs that are currently available from NMIs and other government organizations. The table provides details on the supplier, whether the material is available as a dry powder or a dispersion, the measurands and methods used, and property values and uncertainties. A variety of RMs have been produced by private companies and some of these may have been produced following the ISO requirements. For example, Polysciences, Inc. sells polystyrene particles in a range of sizes, including those under 100 nm, with the primary measurand being the mean particle diameter. The uncertainty for these materials is expressed as a coefficient of variation (CV), typically between 2 and 4%, determined using the disc centrifuge (DC) method. Although the traceability of these particles is linked to NIST SRMs (e.g., NIST 1690, 1692, and 1961), it should be noted that these are microbead standards, not nanoscale reference materials. The reported traceability is established through a calibrated and documented measurement chain, although the specifics of validating this traceability to nanoscale standards are not fully detailed. Nano-sized polystyrene (NT02N–NT05N) and silica (DiagNano™) size standards are termed NIST traceable, which commonly implies the measurement of the application- or scope-relevant properties with an instrument calibrated with NIST SRMs, and are also available from Bangs Laboratories and CD Bioparticles, respectively; however, no details on the traceability process are provided. The traceability of a Malvern Panalytical zeta transfer standard (ZTS1240) was established using NIST SRM 1980, a positively charged goethite material. For this material, also details on establishing traceability are missing.

**Table 1:** Currently available certified reference materials and reference materials from NMIs and other government organizations. Additional informative data is noted under “other information.” Refer to the certificates or certification reports for CRMs for details on establishing traceability. The size measurands for each material are provided in the certificates and include diameters, area equivalent diameters, and other shape metrics for microscopy measurements. For indirect methods such as DLS or CLS, the certificate provides information on intensity vs volume vs number weighting, and the specific measurand (e.g., hydrodynamic diameter for DLS, Stokes diameter for CLS). For both direct and indirect methods, the data may be reported as mean, median, or modal values

Composition/form	Product number	Properties (RM, CRM)	Methods	Values ± uncertainty	Other information	Producer
Gold NPs (aq suspension)	301–01–003 301–01–004	Size (CRM)	TEM DLS SMPS	(34.1 ± 1.7) nm, (94.7 ± 2.4) nm (34 ± 2.4) nm, (87.3 ± 2.5) nm (35.3 ± 1.2) nm, (98.7 ± 1.3) nm	Citrate-stabilized Reference and indicative data from SEM, UV–vis	KRISS
Gold nanocubes (aq suspension)	301–01–006	Size (RM)	TEM	58.1 nm, 55.4 nm	Informative data for SEM, DLS, SMPS, UV–vis	KRISS
Gold NPs (aq suspension)	LGCQC5050	Particle number concentration (RM)	sp-ICP-MS	1.47 × 10^11^/g	Citrate-stabilized Indicative values for modal diameter and gold mass fraction ILC data for multiple methods	LGC
Silver, PVP-coated (powder)	SRM 8017	Size (RM)	AFM TEM DLS	70.1 nm (height) 74.6 nm (diameter) 105.6 nm (Z-average)	USAXS volume weighted equivalent diameter, 67.9 nm	NIST
Silver NPs (aq suspension)	BAM-N008	Size (CRM)	SAXS	(5.8 ± 0.5) nm	Distribution width, number and weight concentration	BAM
Silicon NPs (toluene suspension)	RM 8027	Size (RM) Mass fraction Si (RM)	DLS ICP-OES	2.2 nm 6.43 μg/g Si	Other indicative data are available	NIST
Silica NPs (aq suspension)	301–01–002 301–01-001 301–01–005	Size (CRM)	DLS TEM SMPS	(19.5 ± 2.4) nm, (55 ± 2.5) nm, (89.7 ± 2.4) nm (21.8 ± 0.6) nm, (56.7 ± 1.5) nm, - -, -, (90.6 ± 2.7) nm	Reference data for SEM, SMPS, and colorimetric methods	KRISS
Colloidal silica (aq suspension)	ERM-FD100 ERM-FD304	Size (CRM)	DLS CLS EM SAXS	(19.0 ± 0.6) nm, (42.1 ± 0.6) nm (20.1 ± 1.3) nm (33.0 ± 3.0) nm (19.4 ± 1.3) nm, - (21.0 ± 0.7) nm, -	Other indicative values are available 80-nm colloidal silica available	EC-JRC
Silica NPs, bimodal (aq suspension)	ERM-FD102	Size (CRM)	DLS CLS TEM, SEM	(17.8 ± 1.5) nm, (88.5 ± 7) nm (23.9 ± 2.0) nm, (88 ± 7) nm (18.3 ± 1.7) nm, (83.3 ± 2.3) nm	Indicative dimensional parameters are available for DLS, AFM, PTA	EC-JRC
Colloidal silica^a^ (aq buffer)	SRM 1992	Zeta potential (SRM)	Electrophoretic light scattering	(− 58 ± 5) mV	Conductivity data available	NIST
Colloidal silica^a^ (aq suspension)	ERM-FD101b	Size (CRM)	DLS PTA CLS EM SAXS	(89.5 ± 2.3) nm (87 ± 4) nm (87 ± 8) nm (83.5 ± 2.2) nm (82.5 ± 1.8) nm	Other dimensional parameters are available for some methods	EC-JRC
Mesoporous SiO_2_ (powder)	GBW13909 GBW13910	Specific surface area Total pore volume Average pore size	BET Mercury porosimetry Gas adsorption	492 m^2^/g, 310.0 m^2^/g 0.793 cm^3^/g, 0.875 cm^3^/ 6.47 nm, 11.29 nm	Data on most probable aperture also available	NIM
Alumina (powder)	GBW13901	Specific surface area (CRM)	BET	445 m^2^/g		NCNST
Mesoporous Al_2_O_3_ (powder)	GBW13911 GBW13913	Specific surface area Total pore volume Average pore size	BET Mercury porosimetry Gas adsorption	102.5 m^2^/g, 4.73 m^2^/g 0.259 cm^3^/g, - 10.11 nm, -	GBW13914 also has pore size data	NIM
Alumina (powder)	301–03–004	Specific surface area (CRM)	BET	(100.3 ± 2.5) m^2^/g		KRISS
Cerium oxide (powder)	301–03–005	Specific surface area (CRM)	BET	(28.75 ± 0.4) m^2^/g		KRISS
Iron oxide (toluene dispersion)	BAM-N012	Size (CRM)	TEM	(9.1 ± 0.8) nm (diameter) (8.1 ± 0.7) nm (square edge length)	SAXS informative data	BAM
Titanium dioxide nanorods (butanol suspension)	ERM-FD103	Size (CRM)	TEM, SEM	16.1 nm, 54.0 nm (number weighted medians)	Other dimensional parameters are available	EC-JRC
Titanium dioxide (powder)	SRM 1898	Specific surface area (CRM)	SP-BET MP-BET	(53.85 ± 0.70) m^2^/g (55.55 ± 0.78) m^2^/g	Additional data on crystal phase, DLs size, and from ILC	NIST
Titanium dioxide (powder)	BCR-173	Specific surface area (CRM)	BET	(8.23 ± 0.21) m^2^/g		EC-JRC
Zinc Oxide (powder)	301–03-002	Specific surface area (CRM)	BET	(13.51 ± 0.25) m^2^/g		KRISS
Polystyrene spheres (aq suspension)	301–01–012 301–01–013 301–01–008	Size (CRM)	DLS SMPS	(35.7 ± 2.5) nm, (59.7 ± 2.5) nm, (100.0 ± 2.5) nm (36.1 ± 0.9) nm, (58.3 ± 0.7) nm, (102.6 vs 3.5) nm		KRISS
Polystyrene latex NPs (aq suspension)	NMIJ 5721-a NMIJ 5701-a	Size (CRM)	DMA (diameter, distribution width) DLS	(100.5 ± 2.6) nm (2.4 ± 1.0) nm (118.5 ± 1.8) nm	NaN_3_ preservative added Data for other measurands available	NMIJ
Polystyrene NPs (suspension)	GBW(E)120,090 GBW(E)120,091 GBW12019	Size (CRM)	SEM	79.5 nm 72.9 nm 98.3 nm	Additional data available for other methods	NIM
Reduced graphene oxide (powder)	301–03-003	Specific surface area (CRM)	BET	(629.0 ± 21.2) m^2^/g		KRISS
Single-wall carbon nanotubes	SWCNT-1	Trace metal mass fraction (CRM)	ICP-MS INAA	Co (15.9 ± 1.0) g/kg Ni (14.4 ± 0.8) g/kg Mo (7.3 ± 1.1) g/kg Fe (2.2 ± 0.2) g/kg Pb (6.8 ± 0.9) mg/kg	Reference data for other metals, specific surface area, thermal properties and diameter	NRC
Carbon black (powder)	NMIJ 5714-a NMIJ 5715-a	Specific surface area (CRM)	MP-BET	(110.0 ± 7.3) m^2^/g (18.0 ± 1.2) m^2^/g		NMIJ
Boron nitride nanotubes (powder)	BNNT-1	Diameter, distribution width (RM)	AFM	3.1 nm, 1.2 nm	Informative data for length and elemental composition	NRC
Cellulose nanocrystals (powder)	CNCD-1 (CRM, RM)	Mass fraction sulfur (CRM) Z-average (RM) Height (RM)	ICP-AES DLS AFM	(8720 ± 240) mg/kg S 70.0 nm 3.4 nm	Informative data for TEM, AFM length, crystalline fraction, thermal stability Suspension also available	NRC
Anionic liposomes (aq suspensions)	ALPIO-1	Size (CRM) Polydispersity (RM)	DLS	(90.8 ± 1.2) nm (Z-average) 0.054	Phosphate buffered saline + sucrose; data on storage, refreezing	NRC
Anionic LNPs (aq suspension)	ALNP-1	Size (CRM) Polydispersity (RM)	DLS	(123.3 ± 1.5) nm (Z-average) 0.041	Phosphate buffered saline + sucrose; data on storage, refreezing	NRC

Methods: *AFM*, atomic force microscopy; *BET*, Brunauer–Emmet–Teller surface area analysis; *CLS*, centrifugal liquid sedimentation; *DMA*, differential mobility analysis; *DLS*, dynamic light scattering; *INAA*, instrumental neutron activation analysis; *MP-BET*, multi-point BET; *PTA*, particle tracking analysis; *SP-BET*, single-point BET; *sp-ICP-MS*, single particle inductively coupled plasma mass spectrometry; *SEM*, scanning electron microscopy; *SAXS*, small-angle X-ray scattering; *SMPS*, scanning mobility particle sizer; *TEM*, transmission electron microscopy

Suppliers: *BAM*, Federal Institute for Materials Research and Testing, Germany; *EC-JRC*, European Commission Joint Research Centre; *LGC*, LGC Limited, UK; *KRISS*, Korea Research Institute of Standards and Science; *NCNST*, National Center for Nanoscience and Technology, China; *NIM*, National Institute for Metrology, China; *NIST*, National Institute of Standards and Technology; *NMIJ*, National Metrology Institute of Japan; *NRC*, National Research Council Canada

Only materials with certified or reference data are included. CRMs that were released some years ago and are currently either discontinued or out of stock are not included. Materials available from companies are not included

^a^The same colloidal material was used to produce both SRM 1992 and ERM-FD101b

As follows from Table 1, almost all CRMs and RMs have certified or reference data for size or specific surface area. Only several provide other certified or reference values for properties such as surface charge and number concentration. However, additional informative data is often available for other properties. Some of the first RMs were Au and Ag nanoparticles (NPs), produced by NIST and BAM, as well as polymer NPs and carbon nanotubes. However, these materials are no longer available. Except for the latter, these NMs consisted of spherical (or nearly spherical) NPs with relatively narrow size [30] distributions and a low tendency to aggregate in dispersion or when deposited on substrates for measurements with imaging methods. Recently developed CRMs and RMs increasingly exhibit deliberate deviations from these ideal properties such as non-spherical shapes (Au and iron oxide nanocubes [30] or TiO_2_ rods [31]) and bimodal size distributions (silica NPs [32]) or are made of materials that are more relevant to current applications such as lipid nanoparticles (LNPs [33]) or cellulose nanocrystals (CNCs [34]).

### Methods utilized for RM certification

The certified or reference values for CRMs or RMs in Table 1 were determined by a selection of the methods for NP size measurements as summarized in Fig. 1, e.g., transmission or scanning electron microscopy (TEM, SEM), atomic force microscopy (AFM), small-angle X-ray scattering (SAXS), dynamic light scattering (DLS), or, less commonly, centrifugation methods such as centrifugal liquid sedimentation (CLS). These sizing methods provide values for method-specific (operationally defined) measurands, which are meaningful only within a clearly specified measurement procedure. For selecting the most appropriate method(s) for a specific purpose, the measurement principle of the respective method, its sensitivity, calibration, and validation must be always considered as well as sample properties and sample preparation. Microscopy methods such as TEM, SEM, and AFM provide measurements that are directly traceable to an SI unit via calibration of the microscope. They typically provide images of individual particles on a solid support and yield number-based particle size distributions. Other sizing methods such as DLS, CLS, and SAXS are indirect, ensemble methods that are based on a physical model. In such cases, the size is determined from a calculation or model, and the measurand is specific to the method [35, 36]. The different measurands provided by the various direct and indirect methods result in different sizes for different methods for most nanomaterials, so that a direct comparison is typically not possible. Furthermore, the sizing methods require different strategies for sample preparation and differ in their sensitivity to the presence of aggregates or agglomerates.

### Traceability

As previously stated, the development of any CRM requires that the characterization of the certified property be metrologically traceable, such that the result can be related to a reference through an unbroken chain of calibrations. The traceability determination for nanomaterials is similar to that for any operationally defined measurand, with the method defining the measurand, and the traceability requiring that all relevant influence factors are calibrated. The parameters that must be calibrated will vary depending on the method. Methods for establishing traceability of the methods commonly used for CRM characterization are discussed below. In addition to instrument calibration, other relevant factors include traceability of weighing, calibration of standard equipment and glassware, reagent purity, and usage of appropriate and validated statistical methods for data analysis. For indirect methods, international standards for the models or data evaluation procedures must be included in the traceability statements.

**Fig. 2:**
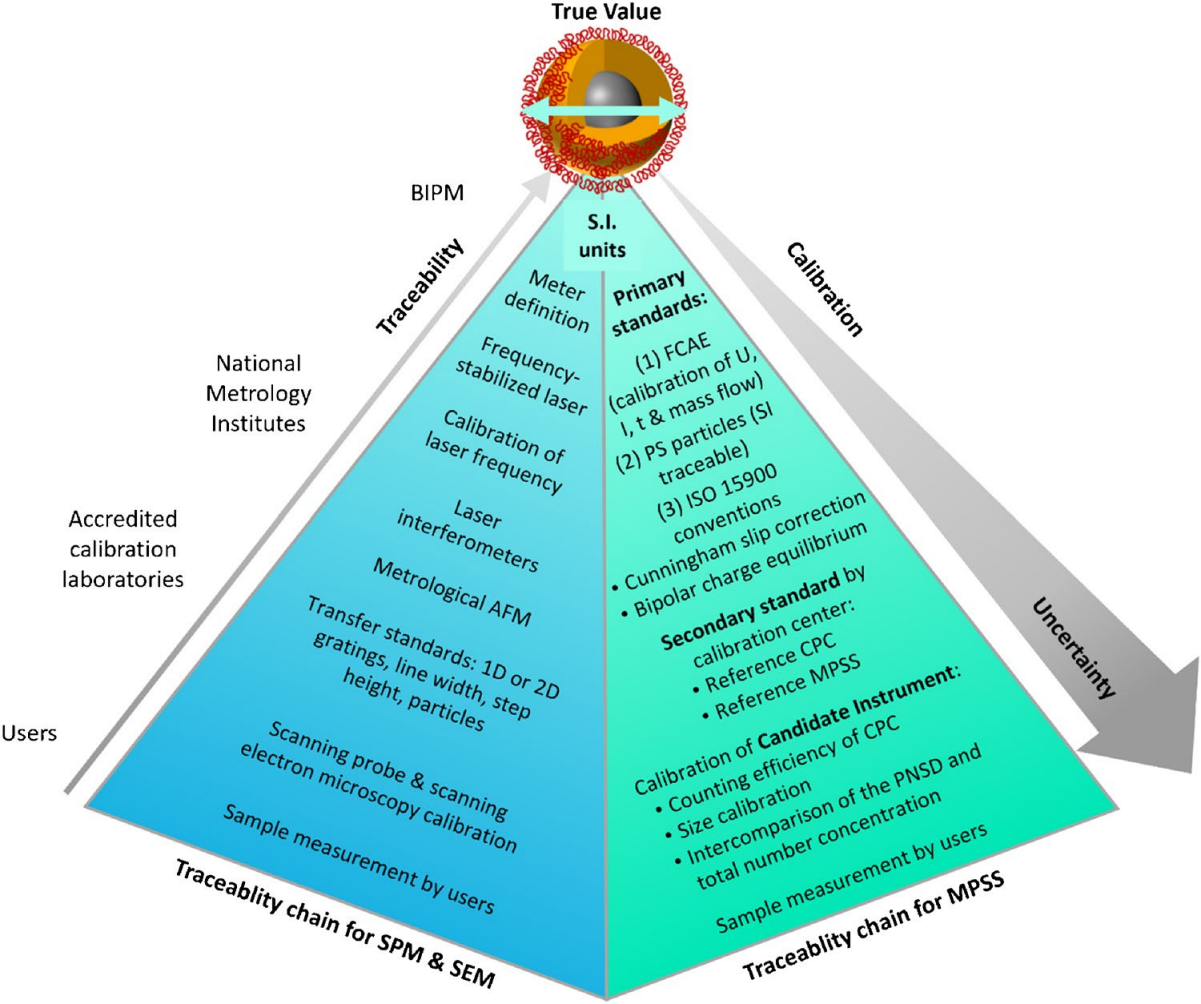
Traceability chain for particle size measurements for nanomaterials by scanning probe and scanning electron microscopy (SPM/SEM, left side) according to reference [83] and by mobility particle size spectrometry (MPSS, right side) according to reference [45]. FCAE, Faraday Cup Aerosol Electrometer; CPC, condensation particle counter; PNSD, particle number size distribution

Traceability can be established for the majority of commonly certified NP measurands summarized in Table 1 (more details are given in the respective RM certificates or certification reports). Microscopy techniques provide traceable values if the respective instruments were calibrated with CRMs such as one- or two-dimensional gratings, step height or line-width standards, or particulate materials (see Fig. 2, left side for an example of traceable particle size measurements for a nanoscale CRM) [37]. For TEM, direct traceability to the SI unit meter is established via the lattice constant of Si [38]. For microscopy measurement, the measurand is the diameter or equivalent circular diameter for spherical or approximately spherical particles and mean, modal, or median values are provided. For scattering methods like SAXS, traceability of the scattering vector can be accomplished through the wavelength of the used radiation and calibration of the used equipment including the detector pixel sizes and distances between sample and detector [39]. All other parameters and models needed must be defined according to the ISO standard. For DLS, traceability is determined by calibration of the instrument clock and temperature sensor and use of the viscosity determined via the calibrated temperature sensor; the instrument function should also be validated using a CRM. The equivalent hydrodynamic diameter is determined as prescribed in the ISO standard [40]. This case falls under a commonly encountered traceability pathway, particularly in chemical or biological measurements: traceability is established to a measurement scale (the SI length scale in this case) via a specific measurement method. As ISO standards can cover several valid strategies for data evaluation that can lead to different results, certificates should specify the used methods, constants, and models. For example, the characterization study of ERM FD304 by DLS limited the used data to the cumulants method for analysis of the correlation function and excluded other evaluation algorithms although they are covered by the ISO standard. Similar issues affect CRMs characterized by centrifugal liquid sedimentation (CLS) yielding the intensity-based modal Stokes diameter that is operationally defined by ISO standards [41, 42]. Here, the characterization studies were limited to centrifuges with a specific detection principle or setup [32, 43]. The traceability of the CLS values is established via calibration with a CRM by the instrument manufacturer as the input parameters of the Stokes equation are usually not known and can be subject to changes during measurement. Also, MPSS provides an electrical mobility diameter which requires calibration of the particle sizing unit with a CRM in addition to the calibration of the counting unit (Fig. 2, right side) according to the ISO standards for instrument calibration [44] and determination of the number-based particle size distribution (PSD) [45, 46].

If a physicochemical property other than particle size is certified, metrological traceability needs to be independently established for this feature. For example, nanoscale CRMs, which are provided as powders, are often characterized for their specific surface area (SSA) using the gas adsorption Brunauer, Emmett, and Teller (BET) method. This provides traceable values if pressure, volume, and mass measurements are traceably calibrated. Additionally, the BET model should be included in the traceability statement [47]. However, none of these RMs is certified for the volume specific surface area (VSSA), which is closely related to particle size and frequently recommended as a measurand for NM size [48]. Particle number concentration can be determined by single particle inductively coupled mass spectrometry (sp-ICP-MS), which is traceable to the SI unit kilogram by gravimetric determination and calibration of all relevant measurement steps such as sample mass flow or transport efficiency [49]. For traceable particle number concentration measurements by SAXS, the scattering intensity must also be traceably calibrated [50, 51].

## Production and certification of nanoscale RMs

The general requirements for the competence of RM producers are defined by the ISO standard 17034 [52]. ISO 33405 provides detailed recommendations for the production and certification of RMs and specifies methods for material selection, determination of homogeneity and short- and long-term stability,^2^ establishing metrological traceability, and assignment of property values with uncertainty budgets [53]. The latter is covered by the Guide to the Expression of Uncertainty in Measurement (GUM) [54].

**Fig. 3:**
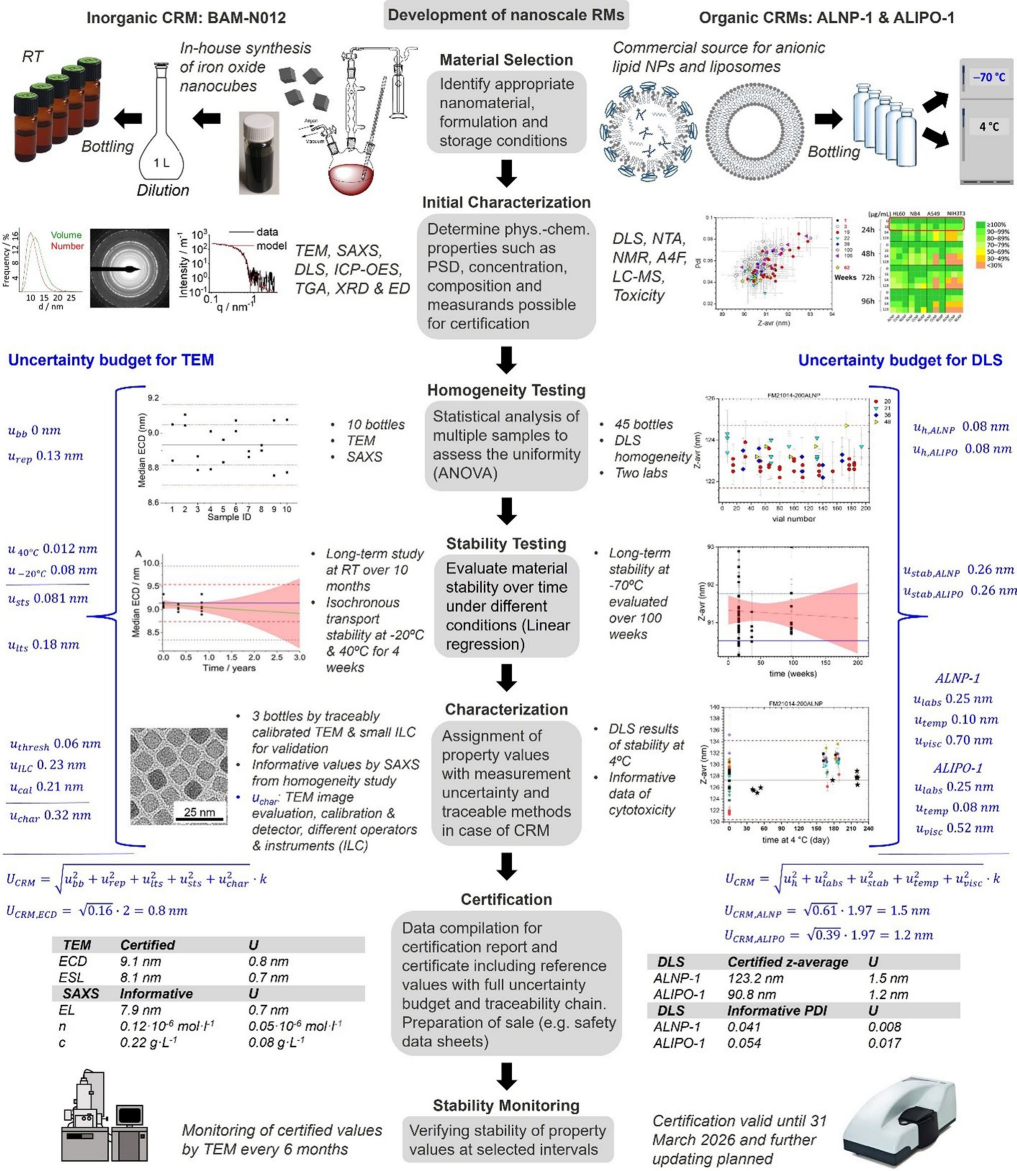
Steps of the production and certification of nanoscale CRMs illustrated by an inorganic and organic example: iron oxide nanocubes (BAM-N012, left side) and organic, lipid-based NMs (ALNP-1 and ALIPO-1 by NRC, right side). Data is taken from references [30, 33, 65]

Figure 3 highlights the key steps of the production and certification of CRMs for recent examples of complex, functional organic and inorganic materials: the certification of the edge length of iron oxide nanocubes by BAM (BAM-N012) [30] and the hydrodynamic diameter of anionic liposomes and LNPs by NRC (ALPIO-1, ALNP-1) [33]. These examples produced by BAM and NRC are cases for which we can most readily illustrate the certification procedures and data evaluation methods. Production of a nanoscale RM requires first consideration of its area of application or scope and the required stability and shelf life as prerequisites to select an appropriate NM and storage conditions. Then, the physicochemical property to be certified and the respective characterization method(s) must be specified along with a target range and uncertainty for the property value of each method. The starting material can be either a commercial NM or a material synthesized in-house by the RM producer. The batch size should be sufficient to obtain enough single units for the certification studies, post-certification stability monitoring, and RM supply. Commercial products are generally available in larger quantities than NMs produced by lab-scale syntheses. In-house synthesis has, however, the advantage that the NM composition is exactly known, including all surfactants, surface ligands, by-products, and possible contaminants. This can facilitate RM reproduction and can sometimes ease the interpretation of measurement results.

Depending on the NM and the intended use, nanoscale RMs are provided as liquid dispersions or solid powders. A colloidally stable dispersion facilitates homogeneous bottling whereas homogeneity across all units can be challenging for an NM powder that contains agglomerates or aggregates. The NM, its dispersant, and other additives also determine appropriate storage conditions of the bottled units of the candidate RM as well as recommended transport conditions. While inorganic or polymer NMs are typically stored at room temperature or 4 °C, since lower temperatures or freezing may lead to aggregation, organic materials such as liposomes require storage at − 70 °C for long-term stability [22, 33]. Lyophilization can also be employed to generate RM units that are long-term stable. In addition, other material-specific factors such as protection from light, oxygen, and/or humidity should be considered as well as characterization method-specific requirements on NM concentration and purity. Biomedical use additionally demands sterile and/or endotoxin free bottling or sterilization of the bottled units [55].

### Homogeneity studies

Subsequently, the homogeneity of the properties to be certified must be examined across all bottled units. Therefore, typically, at least 10 units or the third root of the total bottle number are randomly chosen for analysis from defined subgroups of all units. Trends of the investigated property/properties that reflect the bottling sequence or measurement sequence can be identified by linear regression. RM homogeneity is analyzed by ANOVA to estimate the contribution of possible between-bottle heterogeneity to the uncertainty budget [55]. For colloidally stable NM dispersions, the variation between units is typically not high, often less than the variation due to the repeatability of the measurement method. For methods requiring single particle measurements or lengthy data evaluation as for microscopy methods, the large number of samples and replicates is challenging. In such cases, an alternate method for assessing homogeneity can be selected but must be sensitive to the presence of agglomerates and other sources of sample heterogeneity.

### Stability studies

Some NMs are inherently unstable with changes due to particle dissolution, decomposition of stabilizing ligands (e.g., oxidation in air), Oswald ripening, agglomeration, and aggregation over time. However, not all characterization methods are equally sensitive to such aging effects. Also, the degradation or detachment of additives or surface ligands, revealed by some NMs with coordinatively bound surface ligands upon dilution, can complicate measurements. This is particularly challenging for microscopy techniques. Long-term stability studies to assess RM shelf life under defined storage conditions can be performed either by periodically examining several bottled units (classical design) or by an isochronous approach [56], whereby all samples are measured at the same time (repeatability measurement conditions). Both procedures have been used for the development of nanoscale RMs [30, 31]. The former classical approach requires reproducible measurement conditions over the entire study (intermediate measurement conditions) that can be hampered, e.g., by instrument aging and drift, to be considered. The latter isochronous approach excludes such contributions but requires the selection of adequate storage and reference temperatures for aged samples. This is not always possible. Also, the investigation of a large number of samples at the same time is not feasible for all methods. The isochronous approach is also used for the determination of the short-term or transport stability, mimicking conditions found during transport of the RM to the user, as well as of other external influences like temperature, humidity, or gamma radiation for sterilization. Linear regression is typically employed to analyze the stability data for possible trends and to determine the respective uncertainty contributions. The presence of statistically or technically significant trends in RM stability requires consideration of degradation in the uncertainty budget or assignment of time-dependent values. The expected shelf life can be estimated by defining an acceptable range, typically + / − one-third of the expanded combined uncertainty, for the property value and determining when one of these limits intersects with the 95% confidence interval of the predicted values. This helps to define suitable intervals for stability monitoring after certification.

### Assigning property values

Assigning property values to an RM requires a characterization study that either involves an ILC of several qualified laboratories or involves characterization by one laboratory with a reference procedure that meets the criteria specified in the ISO standard [53]. For the latter approach, confirmation measurements by an independent procedure are recommended. For example, the certified edge length of BAM-N012, measured with a traceably calibrated electron microscope, was validated by comparison with two different microscopes and operators as well as by SAXS as an independent sizing method [30]. All reference values should be good estimates of the true values and all ILC data used for the determination of certified values must be traceable to the same reference. For ILCs, proper calibration of instrumentation used can be ensured by measuring a suitable QC sample, e.g., another CRM [32].

### Uncertainty budget

**Fig. 4:**
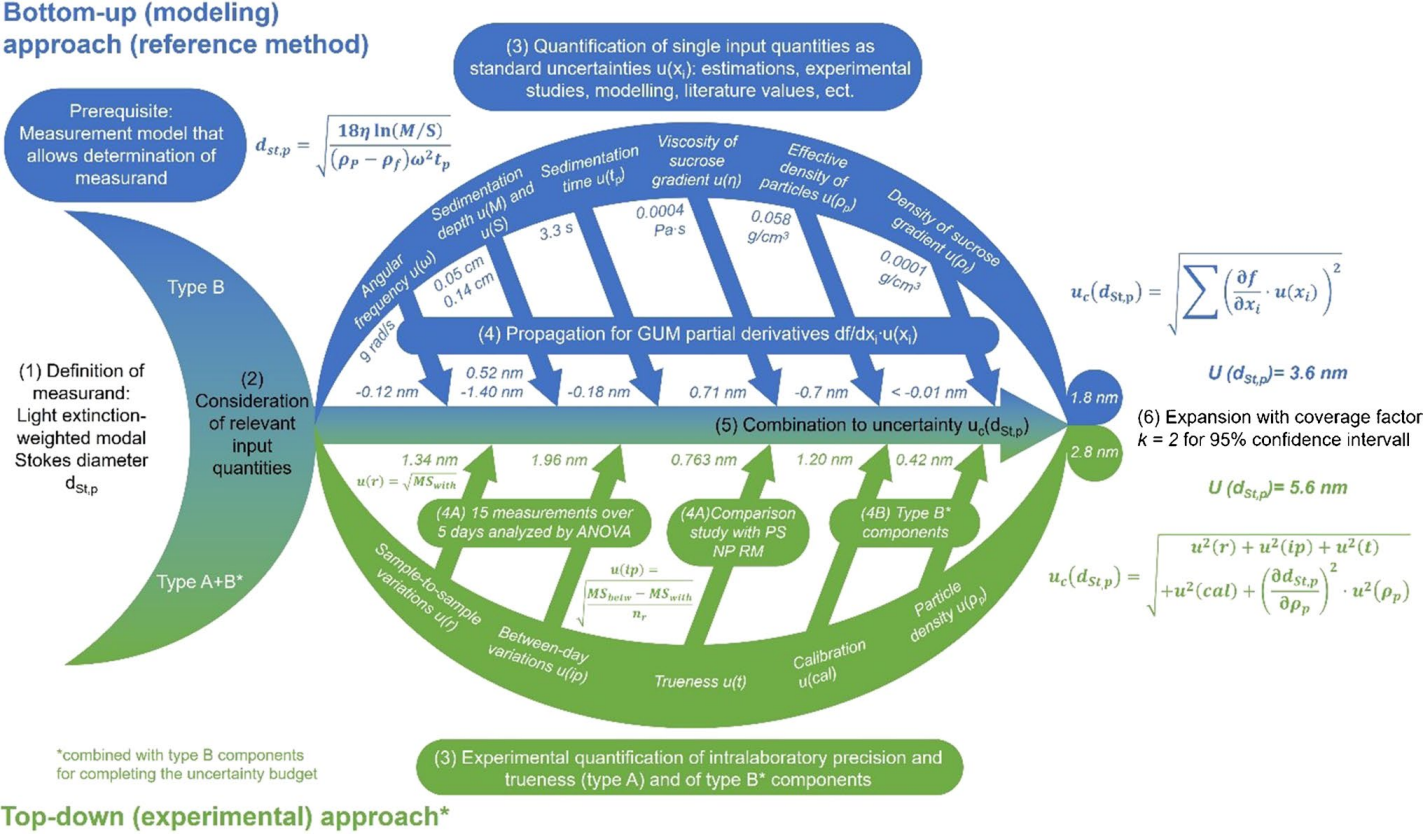
Bottom-up (blue) and top-down (green) approaches for the compilation of an uncertainty budget for CLS measurements. Both approaches were used for single measurements of the SiO_2_ CRM ERM-FD304 according to references [84, 85]

The characterization uncertainty is typically quantified either by experimental determination of the precision and trueness of the respective method (top-down approach, ILC) or by a combination of all individual contributions of the measurement procedure (bottom-up approach), assuming an unambiguous relationship between the property value and all influencing parameters as illustrated in Fig. 4. Therefore, all potential sources of uncertainty in a method must be identified, quantified, and combined according to uncertainty propagation rules. If the unweighted mean of an ILC is used as the property value, its standard deviation corresponds to the uncertainty for the characterization method. The complete uncertainty budget necessary for the certification of a property value is calculated from the contributions of the homogeneity, stability, and characterization studies as shown in Fig. 3 for the two CRMs selected here and is expanded with a coverage factor *k* to reach a certain level of confidence.

## ILCs on NM properties

ILCs are of considerable importance for the validation of physicochemical and analytical methods, including an assessment of achievable uncertainties, and can be used to characterize candidate RMs and to assess laboratory proficiency. Moreover, samples characterized in ILCs with known homogeneity and stability statements can play an important role as quality control samples for the measurands assessed in the ILCs. Many standards development organizations require an ILC to provide pre-normative data on the method prior to publishing a standard. Additionally, property values for RMs can be certified in an interlaboratory study provided that the participants are from qualified test laboratories. Table 2 summarizes selected published ILCs, which contributed to method validation and RM characterization/certification. Some ILCs have only been used to provide data that are summarized in reports or included in Annexes to a standards document and are therefore less readily accessible. Slightly more than half of the Table 2 ILCs were led by NMIs, which typically have a mandate and the resources to undertake such studies. The remainder were led by scientists from academia, industry, and government organizations and in some cases were funded by large EU projects, particularly those focused on metrology. Irrespective of the lead organization, almost all of the ILCS noted in Table 2 had participants from both NMIs and other organizations. In most cases, the ILC was designed to validate specific methods or combinations of methods and to determine the achievable uncertainty across laboratories with differing levels of expertise. However, several JRC-led ILCs were used to provide certified property values for CRMs, as noted in Table 2.

**Table 2:** Selected literature examples of interlaboratory comparisons for NM characterization

Property	Method	Material	Measurand^a^	Reference
Particle size distribution, surface charge	DLS Surface charge	Polystyrene NPs CeO_2_ NPs	Z-average Zeta potential	[86]
Particle size distribution, surface charge	DLS CLS EM SAXS Electrophoretic mobility	Silica NPs	Mean diameter Modal diameter Mean diameter Mean diameter Mean zeta potential	[87]
Particle size distribution	AFM DLS SAXS SEM	Au NPs (10, 30, 60 nm) Silica NPs (40 nm) Polystyrene NPs (50, 100, 200 nm)	Mean diameter	[39]
Particle size distribution	CLS DLS EM SAXS	Silica NPs (20 nm)	Diameter (certification of ERM-FD100)	[43]
Particle size distribution	NTA	Au NPs Polystyrene NPs	Modal particle diameter	[88]
Mass fraction	ICP-MS	TiO_2_ (EC-JRC RTM) in tissue	Mass fraction/organ	[89]
Particle size	AFM SMPS SEM TEM	Aerosol silica NPs (monomodal, bimodal samples)	Mean diameter for monomodal, bimodal samples	[90]
Particle Size	TEM	Au NPs (30 nm)	Mean diameter	[91]
Particle size distribution	sp-ICP-MS	Au NPs (30 nm, 60 nm)	Mean diameter	[92]
Shell thickness	XPS Low energy ion scattering	Au NPs, peptide coated	Elemental composition Thickness of peptide coating	[93]
Particle size distribution	AFM TSEM SAXS	Silica (48 nm, 255 nm, 1 bimodal mixture) Polystyrene particles (100 nm, 147 nm, 1 bimodal)	Mean diameter	[94]
Particle size distribution	AFM DLS PTA SAXS SEM/TEM	Silica NPs (bimodal mixture of 20 nm and 80 nm)	Diameter (certification of ERM-FD102)	[32]
Particle size distribution	TEM	TiO_2_ NPs	Equivalent circular diameter	[95]
Particle size, particle mass fraction and particle number concentration	sp-ICP-MS	PVP-coated Ag NPs in chicken meat	Median diameter, mass fraction, number concentration were not reproducible	[67]
Sulfur content, sulfate half ester contents	ICP-AES Conductometric titration	Cellulose nanocrystals (CNCD-1 CRM)	Mass fraction Sulfate half ester content	[96]
Particle size distribution	TEM	Carbon black	Aggregate shape and size (various descriptors)	[97]
Particle size distribution	TEM	Au nano rods	Max and min Feret diameters, aspect ratio	[98]
Particle size in water and cell culture media	DLS, DCS	Silica NPs, 100 nm Polystyrene, 50 nm, carboxylated and aminated	Hydrodynamic diameter	[99]
Particle size distribution	TEM	Cellulose nanocrystals	Mean particle size, distribution width	[100]
Particle size	PTA	Au NPs, 60 nm	Modal diameter	[101]
Particle size distribution	ICP-MS TEM	TiO_2_ NPs (E 171) TiO_2_ in candy and chewing gum	Mean particle diameter (various other parameters)	[102]
Particle size distribution	DLS PTA TEM/SEM sp-ICP-MS	60-nm Au NPs 40-nm BaSO_4_ TiO_2_ NPs in sunscreen and toothpaste	Mean particle size	[103]
Particle size distribution	AFM	Cellulose nanocrystals	Mean particle size, distribution width	[104]
Particle size distribution	SEM/TEM	TiO_2_ nanorods	Certification of ERM-FD103	[31]
Particle number concentration	CLS PTA SAXS sp-ICP-MS UV–vis spectroscopy	Au NPs (LGCQC5050 RM)	Particle number concentration	[51]
Thermal properties	TGA	Graphene	Mass content of oxygen, carbon	[105]
Particle size distribution	AFM	Graphene oxide	Mean thickness	[106]
Particle size distribution	AFM SAXS TEM	Bimodal Au NPs, bimodal silica NPs, bipyramidal TiO_2_, Au nanocubes	Diameter Height	[107]
Particle mass, particle number concentration	spICP-TOFMS	Pt NPs, 50 nm and 70 nm	Mass, number concentration	[108]
Sample preparation methods	TOF–SIMS	TiO_2_ NPs, 19 nm	Sample quality	[109]

^a^Size measurands vary depending on technique and analysis method; typically, they are reported as mean or median diameters, intensity or number weighted; for irregularly shaped NPs, an area equivalent diameter is provided for microscopy. Consult the reference for details for specific examples

A number of ILCs have been administered by the Versailles Project on Advanced Materials and Standards (VAMAS), an international organization that supports trade in products dependent on advanced materials through international collaborative projects providing harmonized measurements, testing, specifications, and standards [57]. VAMAS has technical working areas on NP populations, graphene, and related 2D materials, and surface chemical analysis, which all include NM relevant ILCs and other NM characterization ILCs that are currently in progress are identified on the VAMAS web site. VAMAS specifies criteria for ILCs for participants, study design, protocol development, and proficiency tests [58]. Other organizations have somewhat different ILC guidelines. For example, ASTM requires ILCs for precision and bias statements for their standards and also recommends the use of RTMs or RMs [59, 60].

Also, key comparisons among NMI members of BIPM CCQM provide data to demonstrate NMI competence in specified techniques relevant for NM characterization and certification. The previously mentioned new task group on particle metrology is likely to undertake additional projects that will contribute to standardization in nanotechnology. For example, recently, a pilot project on particle number concentration was organized by CCQM for 30-nm AuNPs (Table 1, LGCQC5050) in parallel with the VAMAS ILC [51] on the same topic (Table 2), utilizing the same sample. This ILC among NMIs focused on evaluating the accuracy of the methods used and additional validation of the assigned values for the RM. Both VAMAS and CCQM ILCs provided normative data in support of an ISO technical specification on methods to assess particle number concentrations [61].

ILC data also frequently support OECD guidance documents. Some of these ILCs are focused on a single technique (e.g., volume specific surface area using BET), but often use different types of NMs [62] while others combine several characterization methods with a set of different NM samples. For example, a recent study of seven NPs and four nanofibers tested nine different characterization methods including particle counting and ensemble methods and had about 40 participants [63]. This resulted in a test guideline that is more general than an international standard and lacks sample specific details and protocols that are needed to ensure repeatable and reliable data, thereby leaving room for interpretation.

## Current needs and gaps

There are still several gaps in the available RMs and CRMs summarized in Table 1 regarding the need for reliable characterization of application-, regulatory-, and safety-relevant NM properties. First, most of the current RMs are relatively simple, spherical, and monodisperse NMs that avoid the challenging issues of polydispersity and aggregation, which are, however, important for many commercial NMs. Therefore, as previously stated, recent CRM developments have focused on non-spherical NMs, more closely matching “real-world” NMs that have various shapes, e.g., rods, cubes, platelets, and bipyramids, and show bimodal or broader size distributions [30–32, 34]. However, even these materials often still have idealized properties, e.g., a good colloidal stability and a simple composition, facilitating sample preparation and measurement.

Second, there are still many properties that are not addressed by currently available RMs. A 2013 summary of the needs for RMs for environmental, health, and safety (EHS) purposes identified a large list of properties of interest [64], but to date CRMs, RMs, and RTMs for a number of these properties remain to be developed, although validated standard methods are meanwhile available. This slow progress is at least partly due to the time and cost required to produce suitable RMs. The recent release of a suspension of spherical AuNP as RM for particle number concentration as measured by sp-ICP-MS is an important step to extend the range of NM measurands for which RMs are available. This has been only recently complemented by two differently sized lanthanide-based bipyramids with sizes and particle number concentrations traceably characterized by SAXS [65]. However, these two materials are not certified. They were also not characterized with particle tracking analysis (PTA), a widely used lab-based method for determining the number concentration of NMs. As revealed by the earlier mentioned VAMAS ILC of the AuNPs, PTA results suffered from a poor reproducibility across the participants, here revealing a 68% variability and 71% overestimation of the reference value [51]. Other properties, such as functional group (FG) content, FG chemical nature, and NM dispersibility, should be prioritized due to their importance in controlling NM performance in applications and the interaction of NMs with their surrounding and hence, the consequences of inadvertent NM release to the environment and ingestion by organisms. This gap is currently addressed by the EMP project SMURFnano, which targets the quantification of different surface FGs with quantitative NMR (qNMR), optical assays, electrochemical titration methods, and X-ray photoelectron spectroscopy (XPS) and the development of potential RMs with known FG concentration. Moreover, recent OECD work focuses on a guidance document on quantifying surface coatings, involving mass spectrometry techniques as well as XPS.

Another major gap is the lack of RMs and RTMs for NM characterization in complex media such as food, consumer products, nanocomposite materials, and environmental or biological samples or medical formulations. Current RMs fail to reflect the complexity of these materials, making it difficult to ensure consistent and accurate testing across different types of NMs and NM matrices, thus hindering innovation and compliance. There are presently only very few examples for tackling these challenges. Regarding food matrices, an early study supported the feasibility of an RM containing AgNPs in a meat matrix although there were considerable challenges in assessing stability and homogeneity [66]. A later study of AgNPs in chicken concluded that although particle diameter could be reproducibly determined by sp-ICP-MS, there were still issues with the reproducibility for particle number determinations [67]. Related work on silica NPs in soup found that a stable and homogeneous material could be generated but encountered issues with value assignment [68]. Since there are many matrices of potential interest, it will be necessary to prioritize the development of suitable RMs. The potential for EU regulations in various sectors to require determination of properties and amounts of NMs in, for example, food or cosmetics and to consider their life cycle analysis would benefit from availability of RMs in complex matrices. Although some ILCs listed in Table 2 have focused on TiO_2_ and metal NPs in food, RMs in such matrices remain to be developed. The need for such RMs is highlighted by the recent controversy concerning an EU decision to ban the use of food-grade TiO_2_ (E171) based on the 2021 European Food Safety Authority report noting that genotoxic effects related to the fraction of nanoscale particles cannot be excluded [69]. Although this conclusion is supported by food safety committees in some countries, there is mounting evidence that the decision on genotoxicity may be in error [70]. Given the widespread use of TiO_2_, it is critical to have validated methods to characterize this material in representative matrices. There is a strong need for RMs and validated methodologies and protocols and also for RTMs for biological studies aimed at risk assessment and ensuring consistent regulatory practices.

For many applications, NMs are integrated in small amounts into hard or soft matrices such as polymers to create nanocomposites with superior mechanical, magnetic, or optical properties. For example, various organic and inorganic NMs have been exploited to improve the thermal stability, durability, and mechanical and barrier properties against moisture and gas of packaging materials [71], e.g., nanoclays, cellulose nanocrystals (CNCs), biodegradable starch nanocrystals, and silica NPs. Also, other additives such as carbon nanotubes, nanodiamonds, and graphene are used for nano-enabled food packaging [72]. The strong interest in such advanced materials and nanocomposites for packaging or biomedical applications, combined with the significant challenge of characterizing NMs inside a composite material, generates a strong need for suitable RMs and RTMs.

The availability of CRMs, RMs, and RTMs is especially vital for the rapidly advancing field of nanomedicine, where new measurement techniques and novel formulations are regularly emerging. Nanomedicines include liposomal formulations [33], LNP-based vaccines [13], extracellular vesicles [73], metal oxide nanoparticles (MONPs) [1], and nanocrystalline materials like CNCs [3, 74]. The high binding affinity of rationally designed nanomedicine to cancer cells is very advantageous for the targeted delivery of anti-cancer therapeutics, which is also accomplished by liposomal formulations of poorly water-soluble drugs like doxorubicin (Doxil®) that regulate their dissolution or release rates, enhance bioavailability, and reduce cardiotoxicity [75]. Several recent articles have highlighted the need for safety testing of promising nanomedicines [9, 13, 14]. Here, a more widespread use of well-characterized RTMs could help researchers to ensure that their results can be compared to other studies and, in some cases, to determine the effects of physicochemical properties on biological effects, even for not yet fully validated characterization and test methods. This understanding is essential for the safe application and development of new NMs. The development priorities for RMs for nanomedical applications differ based on the product type [13, 22, 73, 76]. For liposome-based nanomedicines, the particle size together with drug payload and chemical composition are crucial parameters, whereas for LNPs, additionally, the surface charge is important. For MONPs, however, agglomeration state, surface chemistry, and crystal phase are fundamental properties to be addressed. This broad range of relevant physicochemical parameters underscores the challenge of creating RMs and RTMs for the varying needs of medical applications. In addition, the nanomedicine industry requires fit-for-purpose RMs, specifically representing clinical formulations available in the market. These needs have been recently addressed by NRC, releasing the first two lipid-based CRMs (see Table 1 and Fig. 3) that also support the establishment of documentary standards. Also, the recently started EMP project MetriNo focuses on the development of RMs and RTMs for nanomedicine with an emphasis on characterization methods for LNPs, liposomes, and MONPs like iron oxide [13].

## Future challenges and possible solutions

In the future, a comprehensive approach is required to address the challenges, needs, and gaps associated with the accurate and reliable characterization of application- and safety-relevant NM properties highlighted in this review. This includes developing more specialized, i.e., fit-for-purpose RMs and RTMs, prioritizing key measurands for which there is a recognized need and user base, harmonizing standardization efforts, increasing the visibility and implementation of standards and SOPs, and improving access to datasets and extensive databases. Thereby, NM industry could enhance the reliability and consistency of NM testing, development, and quality assurance, and regulators could obtain better tools, ultimately advancing the nanotechnology and nanomedicine fields and paving the road for safe applications of innovative NMs in the future. Guidance on how the continuously increasing demand on well-characterized NM can be met could be taken from the concept for Regulatory Preparedness developed for nanosafety assessment [77]. Although current policies may not explicitly prevent the exchange of data or RTMs or test materials, the lack of a structured framework or formal mechanisms can create barriers to widespread and efficient sharing of results. While interested parties can approach ILC organizers for leftover materials, which are sometimes also commercialized (commonly involving stability monitoring), the informal nature of such arrangements can limit accessibility and scalability. Establishing standard procedures such as those used in other fields where unused ILC materials are available could streamline the process. The resource implications for ILC organizers must also be considered as providing ILC materials could, e.g., result in additional costs. Also, current policies should be changed in favor of or towards an exchange of data and RTMS and test materials between multiple stakeholders in collaborative and metrology projects. This could help to speed up the development of new RMs, CRMs, or RTMs. This requires that NMs to be assessed in ILCs where the material stability is sufficient and costs/production efforts are not too high should be ideally prepared in a sufficient batch size for the later purchase by a broader audience. This is sometimes the practice in other areas. 

An alternative approach could be multi-method RMs or test materials that provide reference data for one or more physicochemical properties by various methods, e.g., several particle size measurands combined with surface chemistry or particle number concentration. For many available CRMs, the certification is limited to one measurand with additional data provided at a lower metrological level. Although the certification of multiple measurands and properties is more time-consuming for the RM producer, such a multi-method approach is still less labor-intensive and less costly than producing separate CRMs. Most JRC silica CRMs have been certified for several dimensional measurands, an approach that has been valuable, particularly for laboratories that require multiple measurement methods, for example, a microscopy method and an indirect method for assessing particle size. Such materials are more expensive than other CRMs, but the additional cost is more than offset by a larger number of certified properties. Also, stepwise certification of different properties could be feasible.

The benefit of RMs and test materials for users could be also improved by tailoring these materials more closely to a specific purpose, e.g., by providing NMs deposited on substrates or as a specific formulation for direct measurement to avoid issues associated with sample preparation. It is important to note that the cost of such materials must be offset by a significant demand from the potential user community. In this respect, RTMs and QC samples could present less costly alternatives. In this context, future ILCs should also address such application-relevant measurands and the sources of uncertainty associated with sample preparation which has so far been addressed in a small fraction of ILCs (Table 2). Additional data analysis methods will also have to be developed for methods like XPS or qNMR, to validate results and provide information on typical uncertainties. This in turn can contribute to identifying further challenges in measurement techniques and to improve protocols and fit-for-purpose RMs and test materials.

In addition, the visibility and accessibility of RMs, related reference data, and the corresponding standards and SOPs should be increased, as many companies, especially small and medium-sized enterprises (SMEs), are still unaware of best practices and reference documents. Although standards produced by ISO, IEC, or CEN are very unlikely to be freely available, storing relevant information in selected openly accessible and well-recognized databases and repositories such as eNanoMapper [78] or ZENODO can help to spread this knowledge. This approach should also cover studies providing a valid, accurate, and complete in-depth NM characterization with metadata and reference data meeting the FAIR data policy. Despite the existence of such databases or repositories like eNanoMapper [78] or ZENODO, there are still significant gaps in the availability of metadata, harmonization of reporting standards (quality control), and the use of SOPs [79]. Here, automation, artificial intelligence, and in silico methods such as computational modeling and machine learning could not only develop key components that support read across and grouping approaches in data analysis and NM syntheses [80, 81] but also support property prediction, uncertainty quantification, and ILCs [82].

Overall, standardization of NM characterization and the awareness of the importance of utilizing suitable RMs, CRMs, and RTMs for accurate, reliable, and comparable measurements have considerably matured in the last decade. Nevertheless, there is still a long way to go until standardized methods and approaches for determining all the application- and risk-relevant properties of NMs are at hand, given the complexity of many emerging NMs and relevant matrices. However, the recognized advantages of NMs for many key technologies of the twenty-first century, from optical and sensor technologies to the health and consumer product sector, in conjunction with advances in analytical techniques, grouping concepts, automation and artificial intelligence will speed up these developments in the future.

## Footnotes

1 According to ISO 80004:2023, engineered nanomaterials are designed for a specific purpose or function whereas manufactured nanomaterials are intentionally produced to have selected properties or composition. Incidental nanomaterials are generated as unintentional by-products of a manufacturing or natural process.

2 Short-term stability considers the conditions during transportation to its destination that may affect the property values and the long-term stability the conditions recommended for its storage relevant for its shelf life.
